# Placenta Previa

**Published:** 1876-06

**Authors:** T. Curtis Smith

**Affiliations:** Middleport, O.


SOUTHERN MEDICAL RECORD.

VOL. VI. JUNE, 1876. No. 6

### Original Communications.

### PLACENTA PREVIA.

By T. CURTIS SMITH, M. D., Middleport, O.

There is little need of stating, that, of all the unhappy conditions met with during the course of pregnancy, or in parturition, this is one of the most serious, from the fact that it is unavoidable, and not infrequently compromises the life of the mother, and very often that of the child.

The attachment of the placenta, either partially or centrally over the os uteri is almost necessarily a cause of hemorrhage, as a moment's thought of the vascular attachments of the placenta to the internal surface of the uterus, and the fact that of the continued increase in size of both uterus and placenta, will show clearly. The cause of the placenta being attached over the os internum, is not very clearly demonstrated. Older writers thought (some of them) that this point of attachment was secondary. In other words, that the placenta had been attached at some other part of the internal surface of the uterus, but had become separated from some cause, and by force of gravity had settled to the lowest point and there become secondarily attached. Such an explanation is hardly tenable, and is a theory belonging to an age when the profession scarcely understood the first principles of physiology. But even in this age, the cause of the placenta determining its attachment over the mouth of the uterus, is not very well defined. It seems most probable that it is determined by the ovule gravitating to the lowest part of uterine cavity before becoming attached, after it is received into that cavity.—(Tyler Smith).

The danger to mother and child in these cases is due to the hemorrhage which unavoidably occurs during the progress of gestation or the process of parturition. This hemorrhage is the result of the rupture of one or more of the utero-placental vessels. The rupture is caused by the dilatation of the internal orifice of the cervix. As a rule, the dilatation does not commence prior to about the close of the sixth month, though it may sometimes begin earlier. But when the body and fundus have become quite fully expanded, then dilatation of the cervix at its internal portion begins, and this almost necessarily leads to or causes rupture of some of the utero-placental vessels, and from these the blood flows more or less freely. Sometimes, however, nature is able to accommodate the placenta and its increasing vessels to the gradually expanding cervix, and no hemorrhage occurs until the insensible uterine contractions that precede natural labor become quite strong, or even until real labor sets in. Then the contractions of the body and fundus and the rapidly dilating cervix necssarily lead to rupture of the vascular attachments, and severe loss of blood immediately follows.

It is authoritatively stated that when hemorrhage from placenta previa does not occur until parturition sets in, the hemorrhage is more severe, (Bedford,) and more likely to prove disastrous than where there has been several preceeding losses of blood. On this point I am not prepared to speak, not having had sufficient experience with the different kinds of cases. It is not usual for such cases to run on to the full termination of pregnancy, before any warning of the nature of the case is given by previous hemorrhages. The symptoms of this morbid condition of gestation are too plain to need mention; but a word as to diagnosis may not be out of place. There can be little difficulty in defining the presence, (or not,) of placenta previa, by the taxis, when the os is sufficiently dilated to permit the introduction of the index finger, as in this case the point of the finger will pass directly against the placental mass. A clot, however, may be mistaken for the placenta, and this point is worthy of note. Hemorrhage may occur from other and entirely different causes than the one in question, and still placenta previa be suspected until cleared up by proper investigation. Thus it may arise from syphilitic or cancerous vegetations; from a ruptured vaginal or labial varicose vein; and severe hemorrhage from one lip of the cervix uteri, due to greatly increased vascularity, or from a varicose condition of the cervical veins, which has been, by able practitioners, mistaken for placenta previa. A rare form or cause of profuse hemorrhage, caused by rupture of one of the pudic veins, as well as rupture of a varicose labial vein, has been mistaken for both ante and postpartum hemorrhage from the uterus. Again, hemorrhage from the uterus may occur profusely from separation of a portion of the placenta, when there is no previal attachment. Another point worthy of note, is the fact that but a very small portion of one edge of the placenta may be attached over the internal os, and when broken up may be sufficiently drawn off to one side, so as to no longer be over any portion of the internal os, (*opening of the neck,*) and by a careless digital examination be everlooked, and the point of the finger will only come in contact with the membranes or fœtal head. Still, in such a case the hemorrhage may be very profuse. This cause of the hemorrhage may readily be mistaken for that produced by placental separation higher up.

The prognosis is grave in proportion to the degree of attachment of the placenta, or its approximation to a central implantation We probably might well add, also, in proportion to the time the case is taken in charge by the accoucheur, and his ability to manage the case. According to Schwartz, who collected three hundred and thirty-two cases, there was one death in 3.86, including all varietres. Professor Simpson's statistics show a death-rate of one in 3.6. Trask (prize essay on placenta previa) shows a death rate of one in 3.95. The result in individual cases depends much, of course, on the patient's ability to endure the loss of blood and consequent shock. The mortality is greater in cases where turning is demanded than where it is not. Unfortunately more of these cases give a mal-presentation of the fœtus, than where placenta previa is not present. Fœtal deaths are very frequent.

How shall we manage a case of placenta previa? Undoubtedly this is the most important point for consideration. Few cases, of whatever kind, to which we may be called, are likely to demand a clearer diagnosis of the exact condition and stage of pregnancy or labor than these, and none demand more prompt action, a clear, cool judgment, and a quick discernment of the immediate demands of the case. When the hemorrhage comes on prior to the close of the pregnant term, and the cases are seen before a miscarriage is inevitable, the woman should be placed in bed, on a hard mattress, and kept there to the close of the term. Diet should be as unstimulating as possible, and the bowels kept relaxed. Constipation should by all means be avoided, as straining at stool is very apt to cause a recurrence of the hemorrhage. The bed-pan should be used instead of allowing the patient to rise at the time of voiding the urine or emptying the bowel.

When called at the time labor is progressing, and hemorrhage is going on, if the labor is in the early stage, the os but little dilated, the pains are, or not, very efficient, and there is not room to readily pass the hand into the cavity, nor is the head pressing against the parts firm enough to entirely or measurably control the bleeding, then the tampan is in order, the membrane first being ruptured. This is one of the most valuable means to arrest the free discharge of blood that we can command. It is of especial value where the os is too rigid to permit rapid delivery by turning or otherwise. The tampon, well and thoroughly packed into the vagina, will in these cases check the great flow of vital fluid. It will also act sufficiently as an irritant to the cervix to increase the uterine contraction, thus expediting the labor. But it is claimed that we only check the external flow of blood, while it still goes on internally. If this can be clinically demonstrated, then it is a valid reason why the tampon should not be used. But such is not the facts in the cases above described. A tampon checks the flow by the formation of a salutary clot over the mouth of the bleeding vessels. It is simply impossible for great internal hemorrage to occur when the uterus is already distended with its contents, and the os not at all or but partially dilated. Two things cannot occupy the same space at the same time. The uterus being full, and the vagina also being full to the utmost, there is little room for any fluid to flow into either cavity.

A tampon loosely or improperly applied is worse than useless, as it causes annoyance and irritation to the patient, without effecting the object for which it is used.

One of the best methods of packing the vagina, or tamponing it, is to tear long narrow strips of soft muslin, (say two inches wide); to then introduce a speculum the full depth of the vagina, and pass one end of this strip up against the uterus, then pack with the remaining portion *tightly*, till the whole canal is full, and till not the last vestige can be crowded in. Over this, at the vulva, a T bandage may be applied, or it may be held by an assistant. A portion of the strip or strips of muslin should be left hanging outside the vulva, in order to facilitate its removal. But we have not often a speculum at hand, and often the strips cannot be obtained of sufficient length. If such is the case, bits of muslin—soft—two or three inches square should be crowded in and packed perfectly tight, till it is found impossible to crowd in another particle, and then retained with a T bandage, or held by an assistant. Fine, clean wool, cotton-wool or tow will answer the same purpose, if at hand. I have in this manner so effectually tamponed the vagina that not a drop of blood would escape, and the tampon for half the depths of the vagina would not show a stain of blood. It is true the tampon will absorb some of the blood that flows from the os uteri, but if *tightly packed* it cannot absorb much, and will soon be the means of forming a firm clot about the cervix and bleeding vessels. There are other valuable means of tamponing. The india rubber bag is good, but unfortunately but few practitioners outside of large cities, and many of those in them, have not the pleasure of possessing this and other like facilities. Moreover, if we do possess them, we have not always, or even often, time to spare, in the face of a gushing hemorrhage, to go or send one or a dozen squares to get the needed article; and it seems to me that any practitioner would be very derelict of duty to spend valuable moments, in which a woman's life is ebbing rapidly away, waiting for some favorite appliance in order to check the flow. The emergencies of obstetric practice are often of such a character as not to permit of a moment delay in acting, if we would save the life or lives that are entrusted to our care.

The use of astringents, applied to or near the cervix in the hemorrhage of this class of cases, it seems to me, is sheer nonsense, and worse than useless. They cannot possibly affect the bleeding vessels which they do not reach, and they are quickly washed away by the flow of blood, if that flow is profuse, and it nearly always is. But they have the great disadvantage of exerting their astringent effects on the vaginal mucous membrane and vulva, and thus, without exerting the least effect in checking the hemorrhage, narrow the passage through which we have to operate, and greatly increase the irritation of the vaginal canal and vulva, added to which is the unpleasantly adhesive, nasty sensation which they impart to the fingers of the operator. How long should the tampon remain? This is an important point to decide. Leave it in the vagina till it is crowded well out by the progressing head or breech of the fœtus, or till you have great reason to assure yourself that the os is sufficiently dilated to permit of turning, and deliver by the feet. But I give it as my opinion that where the head or breech is presenting, that it will be safe, as a rule, not to remove the tampon until there is strong evidence that the presenting portion of the fœtus is pressing hard down upon the os, and is crowding down tightly against the tampon, or even crowding it out at the vulva. Whenever the fœtus is crowded down to this extent, it will exert sufficient pressure on the mouths of the bleeding vessels to quite control the hemorrhage. Labor will then go on normally, if the woman has sufficient strength left and the pains are sufficient. Should the head lag in its passage, and the mother or child, or both, seem likely to be sacrificed by the delay, then the forceps should be resorted to without unnecessary delay.

Where it is possible to rupture the membranes, this should always be done early, as it permits the presenting portion of the fœtus to come down and like a wedge exert pressure upon the open vessels with more force and sooner than it could while the membranes remain unbroken. Rupture them if possible before using the tampon.

During the time the tampon is being applied, and afterwards, much can be accomplished in bringing the fœtus down tightly against the os, by external pressure, equally and steadily applied. This can be done by the hands spread out over the fundus, or still better by a broad bandage, or sheet, properly folded and passed around the body, the ends being sufficiently drawn upon by two assistants. These methods are also very valuable means by which to increase the force of the uterine contractions. Cold applications are also well worthy of use in bad cases.

Ergot is a broken stick to lean upon in these cases, if much dependence is put in it, but it may serve as an adjuvant of great value to increase the pains after the tampon is well applied. But if the patient is very weak, and the circulation feeble, its depressing influence over the heart, as well as its tendency to produce cerebral anemia should be considered as great drawback to its use, quinine and whisky, I think, are much more commendable. But more valuable than either of these, in cases of great exhaustion and shock to the patient's system is the free hypodermic use of atropia, from $\frac{1}{30}$ to $\frac{1}{20}$ of a grain. This wil- powerfully stimulate the heart and nerves, and by this means supl ply the great nerve centres with blood and increase the respiration also, thus adding a new link to the tenure by which life is often so slenderly held in these cases.

The child delivered, and the secundines removed, the treatment at once becomes simplified, and this part of the treatment we shall not therefore dwell upon, as every practitioner of intelligence probably knows how to manage a case of exhaustion and shock from puerpural hemorrhage after the uterus is emptied of its contents, and is well contracted. The plan of tearing up all the remaining attached portions of the placenta, when there is previal attachment, advocated by Prof. Simpson, it seems to me, to be of very doubtful propriety. Often it is simply impossible to do so, for the reason that the expansion of the cervix is not sufficient to permit of such manipulation and it is, moreover, often too rigid to permit of sufficiently rapid dilatation to prevent fatal hemorrhage. Besides, I cannot see that tearing up all the utero-placental attachments will close the mouths of the vessels which are left open on the uterine surface internally. We cannot always assure ourselves that uterine contractions will immediately follow of sufficient force to bring the presenting part of the fœtus tightly against the open vessels. The uterus is an organ that is very capricious and uncertain in its action. It may or may not act promptly to stimulus applied, and if it fails to contract, what is there to close the open mouths of the uterine vessels, when the placenta is torn entirely loose from all its attachments.

Again, if the placenta is entirely torn loose from the internal uterine surface, then of course the supply of blood to the fœtus is entirely cut off, and its death is rendered inevitable; whereas, by using a properly applied tampon, or other such means, to check the flow of blood without interrupting the placental attachments, the fœtus is likely, in the majority of cases, to receive a sufficient supply of blood to maintain its life—feebly though it may be—until delivery is accomplished. No one, I believe, expects to succeed in finding the fœtus alive at delivery in all cases where previal attachment is present, but because we may expect its death after very large losses of blood, is no reason why we should recklessly destroy every chance left us to save its life.

I will give two cases of placenta previa occurring in my practice, which will, I think, be typical cases of this class, and their management will illustrate my views of the proper method of managing them under the circumstances in which I was situated.

Mrs. G., æt. 33, sixth pregnancy: During the course of gestation had noticed nothing unusual until within four weeks of the close of her term. At that time there occurred suddenly, and without previous warning, a free gush of blood from the vagina. Being sent for immediately, I saw her in a half hour after the appearance of the first show of blood. She was lying on the bed, pale as death, gasping for breath, very restless, and manifesting great anxiety for her safety ; pulse scarcely perceptible ; there was no perceptible contraction of the uterus. Vaginal examination showed a large clot in the vagina, which I removed. Then passing my finger up to the os, found it was closed and the cervix obliterated. The finger was withdrawn, and all clots and blood removed from about her person that could be without disturbing her position and quietude. Clean napkins were then one after another placed against the vulva, and changed very often at first, but at longer intervals later. Careful watching in this way, and the use of the vaginal taxis an hour later, showed that there was no further hemorrhage beyond a mere stain. During this time, and until she had rallied, her head was kept low, perfect quietude maintained, and she was revived by the use of cold drinks given often in small quant ties. After two hours of waiting and watching there was no indication of an immediate return of flooding, and not a sign of any uterine contractions, and the patient was commencing to rally quite well. After ordering the strictest quiet, an unstimulating diet, directing the use of the bed-pan, instead of getting up, in case it should be needed, left the patient. I saw her frequently during the next twenty-four hours, and visited her for several days. She carried out my directions strictly. After the first two days she began to regain the natural color of the face and mucus surfaces, and soon implored me to let her get up. But to prevent this or any other wish she might have concerning her actions, I had only to remind her of her critical situation and the danger of a return of the flooding. I instructed her to call me at the first intimation of a return of the hemorrhage, or when she felt the first twinges of pain in labor. She went on for one month, when I was hastily summoned with the word that she was in labor and was flooding. I soon reached her bed-side only to find it all true. She was pale, feeble, alarmed, pulse very weak, and the breath coming hard. As usual, her lady attendants were fearfully excited and alarmed. A vaginal examination was made hastily, which disclosed an os open to admit the tip of the finger, tolerably rigid, and the placenta plainly to be felt, latterally attached, probably two inches of its surface attached to the left of the os, while the body was to the right. A head presentation was made out. The uterine contractions were occurring every five to eight minutes, but not strong. I at once ruptured the membranes, and tamponed the vaginal canal very tightly with small pieces of soft muslin, as before mentioned, after which not a drop of blood escaped externally till the tampon was removed. The patient's head was kept low, a firm bandage—broad—was passed over the fundus and body of the uterus and brought under the loins, the ends of which were given to an assistant on either side of the bed, with directions to keep it drawn as tightly as the patient would permit. Whisky and quinine were used quite liberally, ammonia was inhaled, cold drinks ad libitum After free emesis she began to rally, the treatment being continued. In two hours from the time the tampon was applied her pulse returned with moderate force, there was some color again in her lips and face, and the countenance had lost its wild, anxious appearance; the uterine contractions were now becoming quite forcible. Soon after this it was evident that the tampon was being crowded down tightly against the perineum, when it was then gradually removed; and the head was found to be pressing firmly on the bleeding surface. After this there was no further trouble. She was delivered of a feeble, but well-developed, male child, weighing eight pounds, living. I had assured her that she should get through her trouble, and get well, but would make no promises concerning the safety of the child. She was able to resume her domestic duties in three weeks from date of confinement. She was naturally a strong, healthy woman.

What amount of blood this lady lost at each attack of hemorrhage I do not know. In each instance there was a large pool found in the bed, the clothing and bed being saturated so that it soaked through, and dripped quite profusely on the floor beneath. Some of her symptoms of severe prostration, no doubt, were the result of fright, but the loss of blood was very great.

I will take occasion here to remark that the style of introducing a tampon, by the use of a handkerchief, or a large piece of cloth of any kind, is quite useless. It cannot be packed with sufficient firmness to prevent the blood from passing out by the side of or under it. Whenever the outside cloths of a tampon become saturated with blood, or permit the passage of blood around it, and its escape externally, it is evidence that it is not sufficiently and properly applied.

Case II Mrs. H., æt 36, of nervo-sanguinous temperament; of rather poor health generally; has borne children quite rapidly. During her seventh pregnancy, two months prior to the close of the full term, she was awakened one night by feelings of distress, and found she had been flooding very profusely. Some domestic treatment was instituted; the hemorrhage ceased. It recurred again a few weeks later. She continued, however, to do all her housework for a large family to within a few days of the time of her expected confinement. At the time of her expected delivery she was awakened by labor-pains and a profuse hemorrhage. A midwife was called in, who thought she could manage the case without assistance. She continued to minister to the wants af her patient from 3 a.m. to 9 a.m., when she told the husband he must get something from the doctor to stop the flooding, then she could get along all right. The husband came to me to get the necessary medicine. After getting a history of the case, I told him it was not medicine he wanted; it was the immediate attendance of some physician who understood his business; for his wife must be in imminent peril. He insisted on the medicine, and seemed to think they could get along. I gave him ergot to use freely, telling him it would do his wife no good, and that nothing in the shape of medicine would stop the hemorrhage. After his return home the hemorrhage continued till fainting had occurred several times, when, finally becoming alarmed, I was sent for, and saw the patient at 1 p.m. A more distressed-looking woman I have seldom seen. Examination showed a centrally implanted placenta previa, os dilated and soft, no contractions having occurred for some time before my arrival. In fact, the attendants thought at one time that the woman was dead. I told the husband I thought his wife would die, but would strive to save her; asked for council, which was refused. I placed the patient's head as low as possible, gave her, hypodermically, a syringeful of whisky and the one-twentieth grain of atropia as quickly as possible; then put her in position, passed my left hand up through the placenta and membranes, drew down the feet and delivered. As the feet were drawn down, the uterus contracted well, and so continued to do, thus causing me no fear of post-partum hemorrhage. The placenta immediately followed the delivery of the fœtus; the latter being dead of course. (I found on first examination that the midwife, while officiating, had picked the presenting portion of the placenta to pieces, thus increasing the hemorrhage.) For four hours after delivery, (and how long before is not known), there was no perceptible pulse at the wrist, extremities cold, the patient having a death-like appearance, and had to be kept roused to prevent respiration from stopping. Kept head low, applied dry heat, gave stimulant per rectum, per ovum and hyperdermically. During the fifth hour there was some signs of reaction, such as the return of a very feeble pulse at the wrist, increase of warmth, a little color in the lips, respiration deeper, easier, etc. From this date onward there was *slow* but steady improvement; but she was five weeks in bed, and during all the first three could not sit up at all, without the occurrence of sincope, or distressing symptoms of its approach. This was not a case for the use of the tampon. Indeed, dependence on it would probably have led to certain death. So that while I strongly advocate the use of the tampon, when the os is not dilated, or is but partially dilated, or is rigid, I would not commend it when dilatation is sufficient to permit turning, or of using the forceps. The forceps in this case could not have been readily applied, for the head had not commenced to engage, the membranes were intact, and the central attachment of the placenta would have rendered it a great obstacle to their speedy application. I think the fœtus was turned and delivered in less time than it would have taken to apply the forceps.

Would it have been proper to use the tampon, if the case had been seen early? I think it would, for by its use the hemorrhage would have been stopped before the patient became too much exhausted for delivery to have been effected by uterine contraction, and without turning. Certainly the hemorrhage could not have been as profuse early in the labor as we would reasonably expect, otherwise the patient would have died from its effects long before I was called. After seeing to the demands of the case, I gave the midwife a left handed blessing, mingled with as wholesome advice as could, hoping—probably in vain—that it might prove of benefit to her and her patrons in the future. There are many points of interest in the history of placenta previa, and its study, that I have purposely omitted, in order to avoid greatly extending the limits of this paper.

I think it is altogether proper in these cases to use ergot, when the pains are inefficient, after the tampon has been well applied, but the dependence on it to check hemorrhage in placenta previa is worse than useless. To arouse the pain after the hemorrhage has ceased, quinine and whisky are better. But the first demand is, by some means, to control the hemorrhage and then afterwards the choice of remedies to meet other demands can be left for more mature consideration by the accoucheur. One point in the use of ergot in this class of cases that, so far as I know, has, in obstetric practice, been entirely overlooked, i. e., its secondary depressing influence over the heart and nervous system. Patients who take ergot largely, either in ante or post partum hemorrhage, are, according to my observation, longer in rallying than those who do not take it, and are also much more apt to be troubled with nausea and vomiting. If the hemorrhage can be controled without its use, some agent that will promptly arouse the nervous system to action is preferable, for when this is thoroughly and normally aroused, there is not likely to be a want of uterine contraction. As a simple control of post partum flooding—barring all other points—ice s far more efficient and preferable to ergot.

